# Supplementary material for: Roles of Kdm6a and Kdm6b in Regulation of Mammalian Neural Regeneration
Source: Adv Sci (Weinh). 2025 Feb 14;12(16):2405537. doi: 10.1002/advs.202405537 (PMC12021076; doi:10.1002/advs.202405537)
Supplement: Supplementary file 1 — Supporting Information [file ADVS-12-2405537-s001.pdf]

ADVANCED SCIENCE
Open Access

Supporting Information

for *Adv. Sci.*, DOI 10.1002/advs.202405537

Roles of Kdm6a and Kdm6b in Regulation of Mammalian Neural Regeneration

Shu-Guang Yang, Chang-Ping Li, Xue-Wei Wang, Tao Huang, Cheng Qian, Qiao Li, Ling-Rui Zhao, Si-Yu Zhou, Chen-Yun Ding, Rui Nie, Saijilafu, Yu-Cai Hong*, Chang-Mei Liu* and Feng-Quan Zhou**

Extended Data Figure 1

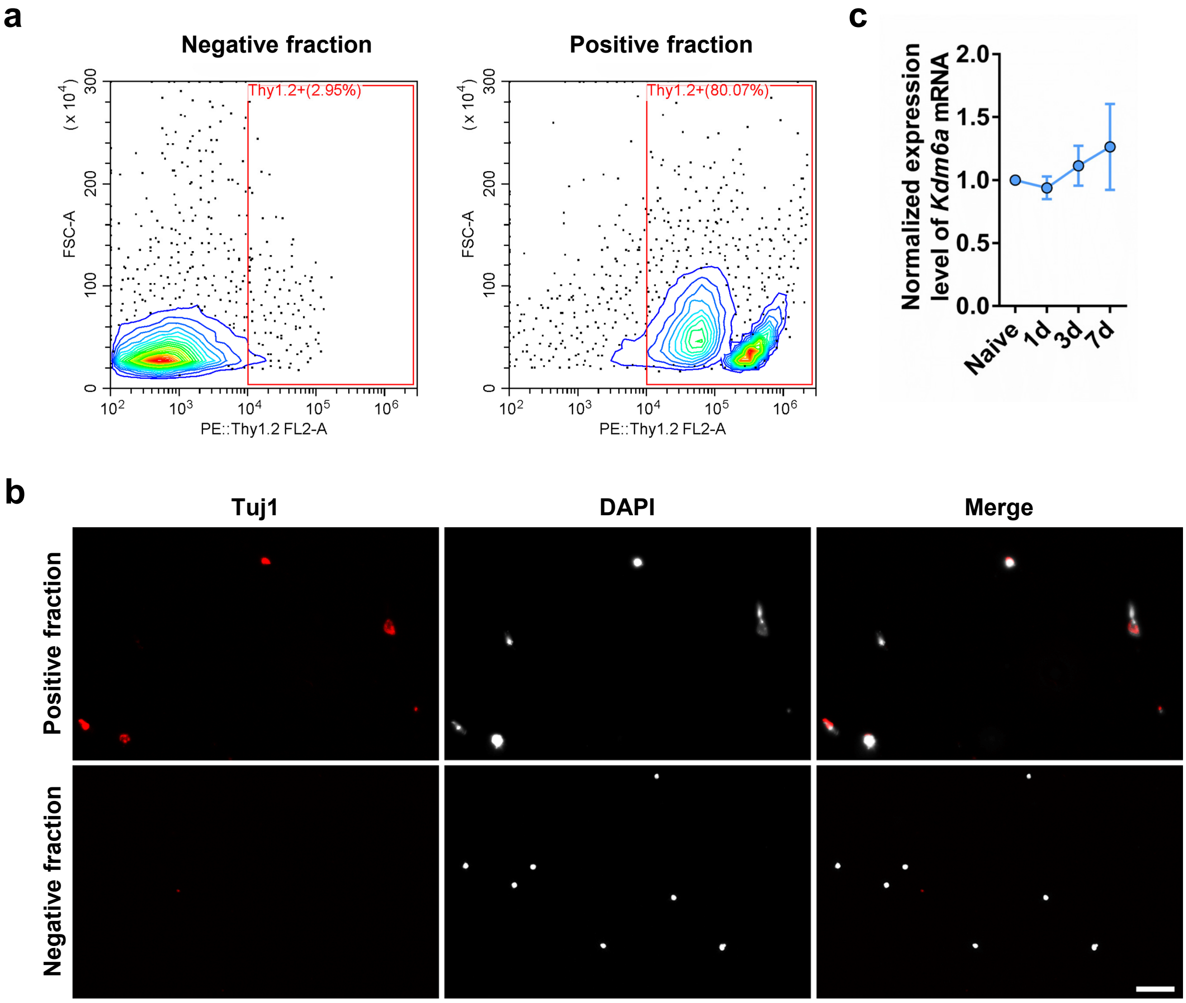

Extended Data Fig. 1 | Unchanged mRNA level of *Kdm6a* in RGCs after ONC, Related to Fig. 2.

(a) Positive selection and depletion of Thy1.2+ RGCs by MACS. Labeled retinal cells with anti-mouse Thy1.2 magnetic particles were separated into negative fraction and positive fraction using a magnet. Thy1.2+ cells in each fraction were shown in the right box (red). Flow cytometric analysis showed that the positive rate of Thy1.2 in positive fraction was 80.8±4.43% (*n*=3 independent experiments). The threshold for Thy1.2+ cells was determined based on the cell distribution in isotype control.

(b) Representative images of Thy1.2+ RGCs after MACS purification. Retinal cells in negative fraction and positive fraction were stained for neuronal marker Tuj1 (red) and DAPI (gray). Scare bar, 50 μm.

(c) Time course of expression of *Kdm6a* transcripts in purified RGCs from naive mice and 1, 3, and 7 days post-ONC by real-time PCR analysis. (one way ANOVA followed by Tukey's multiple comparison test, *p*=0.6631; *n*=3 independent experiments for each condition).

Data are represented as mean ± SEM.

Extended Data Figure 2

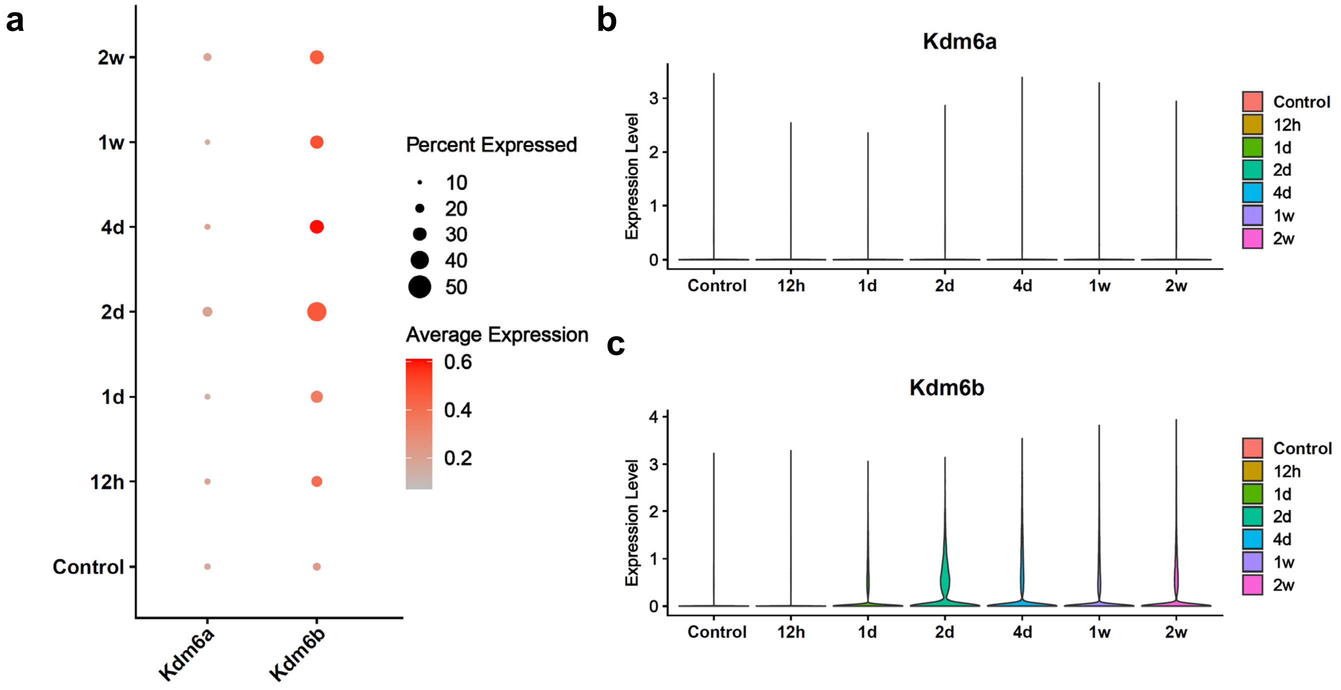

Extended Data Fig. 2 | Changes of *Kdm6a/b* in RGCs after ONC, Related to Figs. 2, 3.

(a) Dot plot showing expression patterns of *Kdm6a* or *Kdm6b* in RGCs at different time points after ONC. The size of each circle is proportional to the percentage of RGCs expressing *Kdm6a* or *Kdm6b* and their intensity depicts the average transcript count within expressing RGCs.

(b) Violin plot indicating the expression distribution of *Kdm6a* within RGCs at different time points after ONC.

(c) Violin plot indicating the expression distribution of *Kdm6b* within RGCs at different time points after ONC.

(a), (b) and (c) obtained from GEO: GSE137398.

Extended Data Figure 3

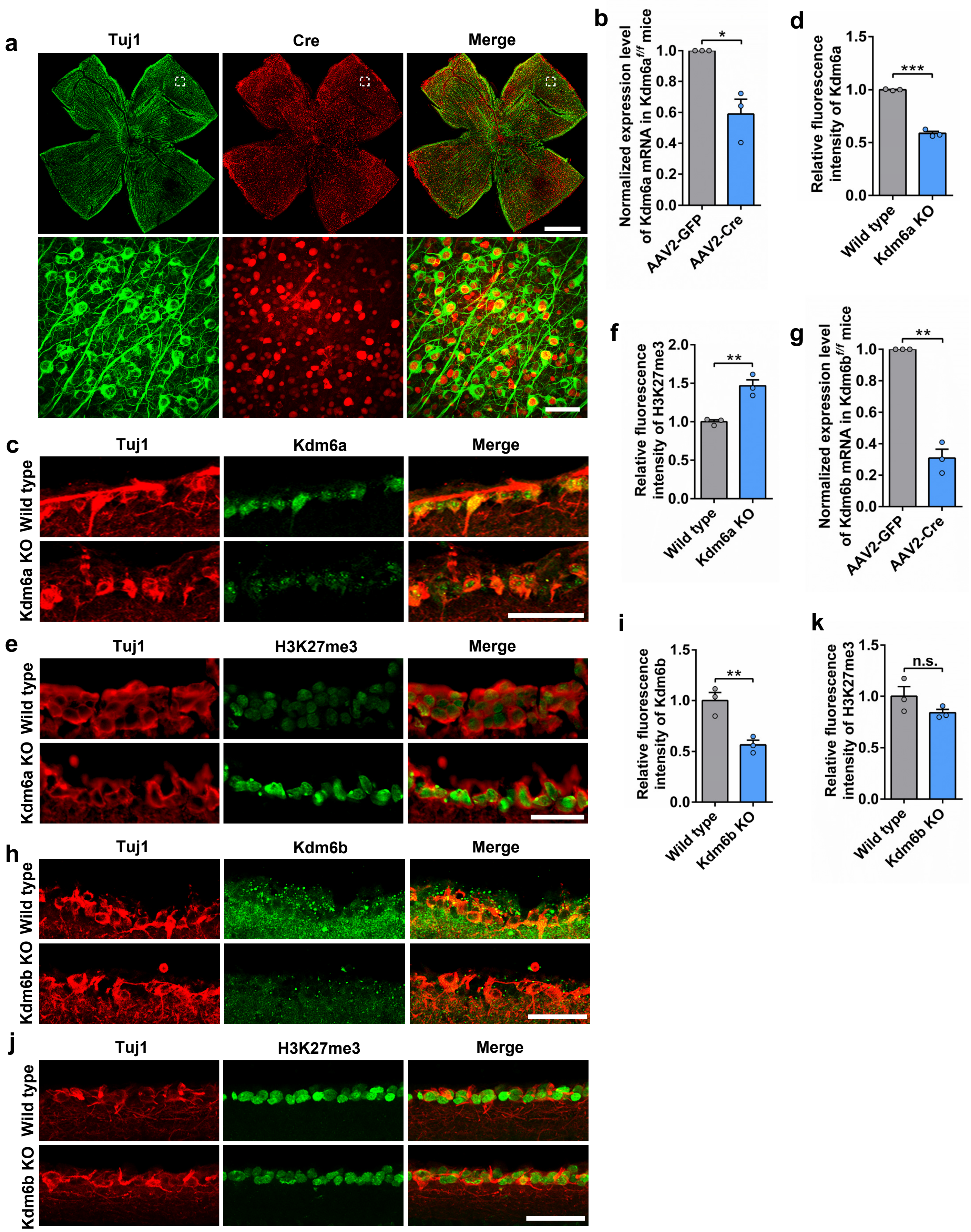

Extended Data Fig. 3 | Deletion of Kdm6a in RGCs triggered significantly increased level of H3K27me3, but not Kdm6b deletion, Related to Figs. 2, 3.

(a) Confocal images of flat mounted retinas showing co-immunostaining of Tuj1 (green) and Cre recombinase (red). The bottom row images show enlarged images of the top row images. The infection rate of AAV2-Cre in RGCs was 88.8 ± 7.56% (*n*=2 independent experiments). Scale bar, 1 mm in top images and 50 μm in bottom images.

(b) Real-time PCR analysis showing mRNA levels of *Kdm6a* in the purified RGCs of $Kdm6a^{f/f}$ mice two weeks after intravitreal injection of AAV2-GFP or AAV2-Cre, respectively (one sample *t* test, *p*=0.0499; *n*=3 independent experiments).

(c) Confocal images of retinal sections showing significantly reduced level of Kdm6a in RGCs of $Kdm6a^{f/f}$ mice injected with AAV2-Cre (Kdm6a KO), compared to wild type mice with AAV2-Cre injection (Wild type). The sections were stained for neuronal marker Tuj1 (red), which labeled RGCs, and Kdm6a (green). Scale bar, 50 μm.

(d) Quantification of relative fluorescence intensities of Kdm6a immunostaining shown in (c) (two tailed student's *t* tests, *p*<0.0001; *n*=3 mice for each condition).

(e) Confocal images of retinal sections from wild type and *Kdm6a* knockout mice (Kdm6a KO) showing markedly increased level of H3K27me3 via deletion of Kdm6a in RGCs. The sections were stained for neuronal marker Tuj1 (red), which labeled RGCs, and H3K27me3 (green). Scale bar, 50 μm.

(f) Quantification of relative fluorescence intensity of H3K27me3 immunostaining shown in (e) (two tailed student's *t* tests, *p*=0.0054; *n*=3 mice for each condition).

(g) Real-time PCR analysis showing mRNA levels of *Kdm6b* in the purified RGCs of $Kdm6b^{f/f}$ mice two weeks after intravitreal injection of AAV2-GFP or AAV2-Cre, respectively (one sample *t* test, *p*=0.0067; *n*=3 independent experiments).

(h) Confocal images of retinal sections showing significantly reduced level of Kdm6b in RGCs of $Kdm6b^{f/f}$ mice injected with AAV2-Cre (Kdm6b KO), compared to wild type mice with AAV2-Cre injection (Wild type). The sections were stained for neuronal marker Tuj1 (red), which labeled RGCs, and Kdm6b (green). Scale bar, 50 μm.

(i) Quantification of relative fluorescence intensities of Kdm6b immunostaining shown in (h) (two tailed student's *t* tests, *p*=0.009; *n*=3 mice for each condition).

(j) Confocal images of retinal sections from wild type and *Kdm6b* knockout mice (Kdm6b KO) showing a slight reduction in the level of H3K27me3 via deletion of Kdm6b in RGCs. The sections were stained for neuronal marker Tuj1 (red), which labeled RGCs, and H3K27me3 (green). Scale bar, 50 μm.

(k) Quantification of relative fluorescence intensity of H3K27me3 immunostaining shown in (j) (two tailed student's *t* tests, *p*=0.1817; *n*=3 mice for each condition). Knocking out *Kdm6b* in RGCs resulted in a mild reduction in H3K27me3 levels, which did not reach statistical significance.

n.s. *p* > 0.05; *, **, ****p* < 0.05, 0.01, 0.001, respectively. Data are represented as mean ± SEM.

Extended Data Figure 4

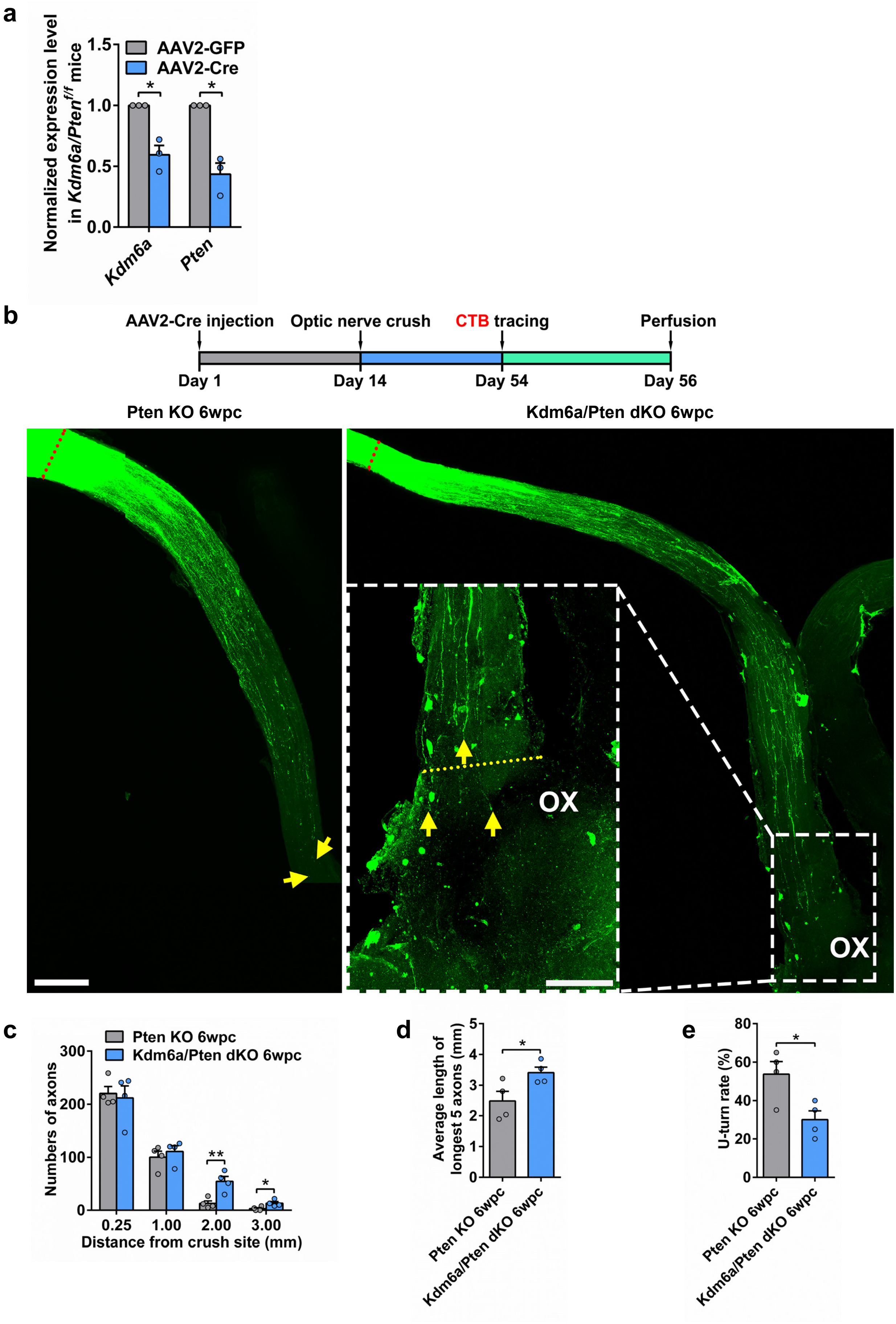

Extended Data Fig. 4 | Additive effects of *Kdm6a* and *Pten* double knockout on axon regeneration observed 6 weeks after ONC, Related to Figs. 5, 6.

(a) Real-time PCR analyses showing mRNA levels of *Kdm6a* or *Pten* in the purified RGCs of *Kdm6a/Pten*$^{f/f}$ mice 2 weeks after intravitreal injection of AAV2-GFP or AAV2-Cre, respectively (one sample *t* test; *Kdm6a*: p=0.0331; *Pten*: p=0.0253; n=3 independent experiments).

(b) Top: schematic time course of optic nerve regeneration model. Optic nerve regeneration was analyzed 6 weeks after the nerve crush, and the fluorescence (Alexa 594) tagged CTB was injected 2 days before collecting the optic nerve. Bottom: representative 2D projected confocal images of whole mount cleared optic nerves of *Pten* knockout (Pten KO 6wpc) or *Kdm6a*/*Pten* double knockout (Kdm6a/Pten dKO 6wpc) mice 6 weeks after ONC. The regenerating axons were labeled with CTB-Alexa 594. The left dashed white box shows enlarged images of areas indicated by the dashed white box on the right. Yellow arrows indicate the distal ends of regenerating axons. Red and yellow dotted lines indicate the nerve crush sites and the optic nerve-chiasm transition zone, respectively. OX indicates the optic chiasm. Scale bar, 200 μm.

(c) Quantification of regenerating axons at different distances distal to the nerve crush site (0.25 to 3.00 mm) in *Pten* knockout (Pten KO 6wpc) or *Kdm6a*/*Pten* double knockout mice (Kdm6a/Pten dKO 6wpc) 6 weeks after ONC (two tailed student's *t* tests, p<0.05; Pten KO 6wpc: n=4 mice, Kdm6a/Pten dKO 6wpc: n=4 mice).

(d) Quantification of the average length of the top 5 longest axons of each nerve in (b) (two tailed student's *t* tests, p=0.0442; Pten KO 6wpc: n=4 mice, Kdm6a/Pten dKO 6wpc: n=4 mice).

(e) Quantification of U-turn rate in (b) (two tailed student's *t* tests, p=0.0250; Pten KO 6wpc: n=4 mice, Kdm6a/Pten dKO 6wpc: n=4 mice; top 20 longest axons were analyzed for each mouse).

*,**p < 0.05, 0.01, respectively. Data are represented as mean ± SEM.

Extended Data Figure 5

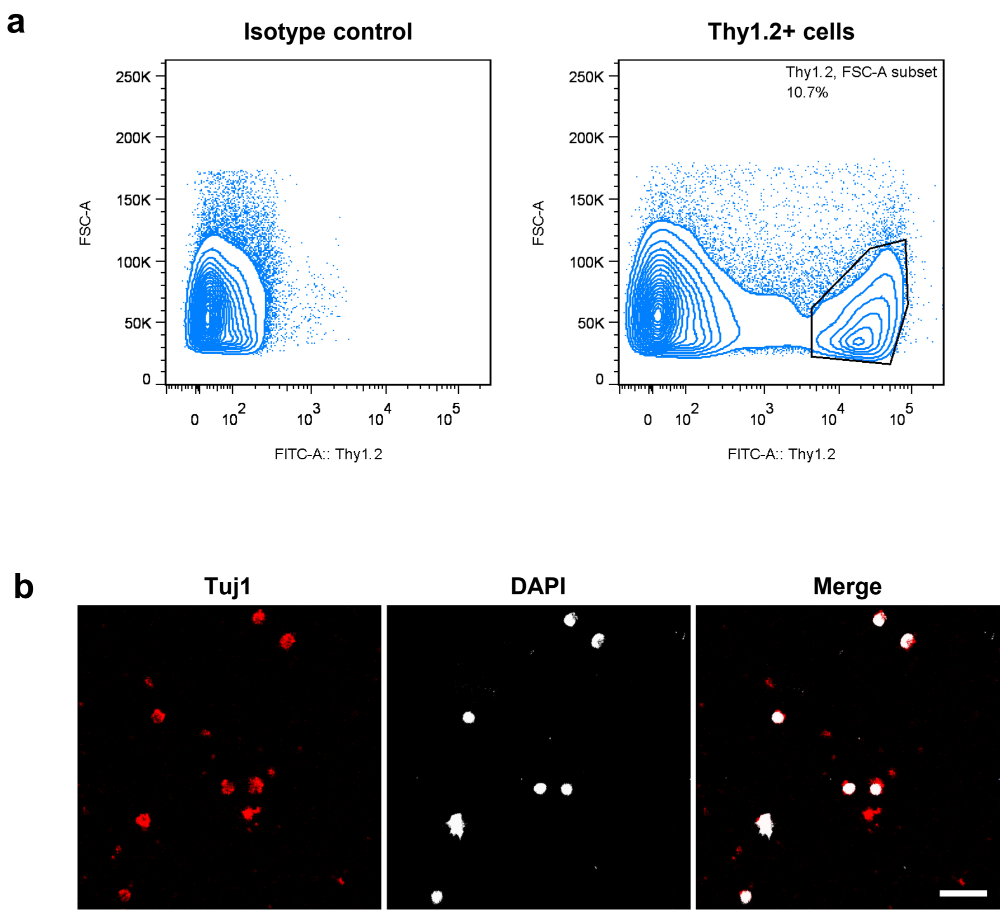

Extended Data Fig. 5 | Enrichment of mouse Thy1.2+ RGCs by FACS for high throughput sequencing, Related to Fig 7.

(a) Representative FACS plots showing RGCs enrichment. Retinal cells labeled with isotype antibody were used as controls to set up the threshold for Thy1.2+ cells. Thy1.2+ RGCs were collected according to isotype control. Cell viability was determined with DAPI staining.

(b) Representative images of Thy1.2+ RGCs after FACS sorting. Purified RGCs were stained for neuronal marker Tuj1 (red) and DAPI (gray). Scare bar, 20 μm.

Extended Data Figure 6

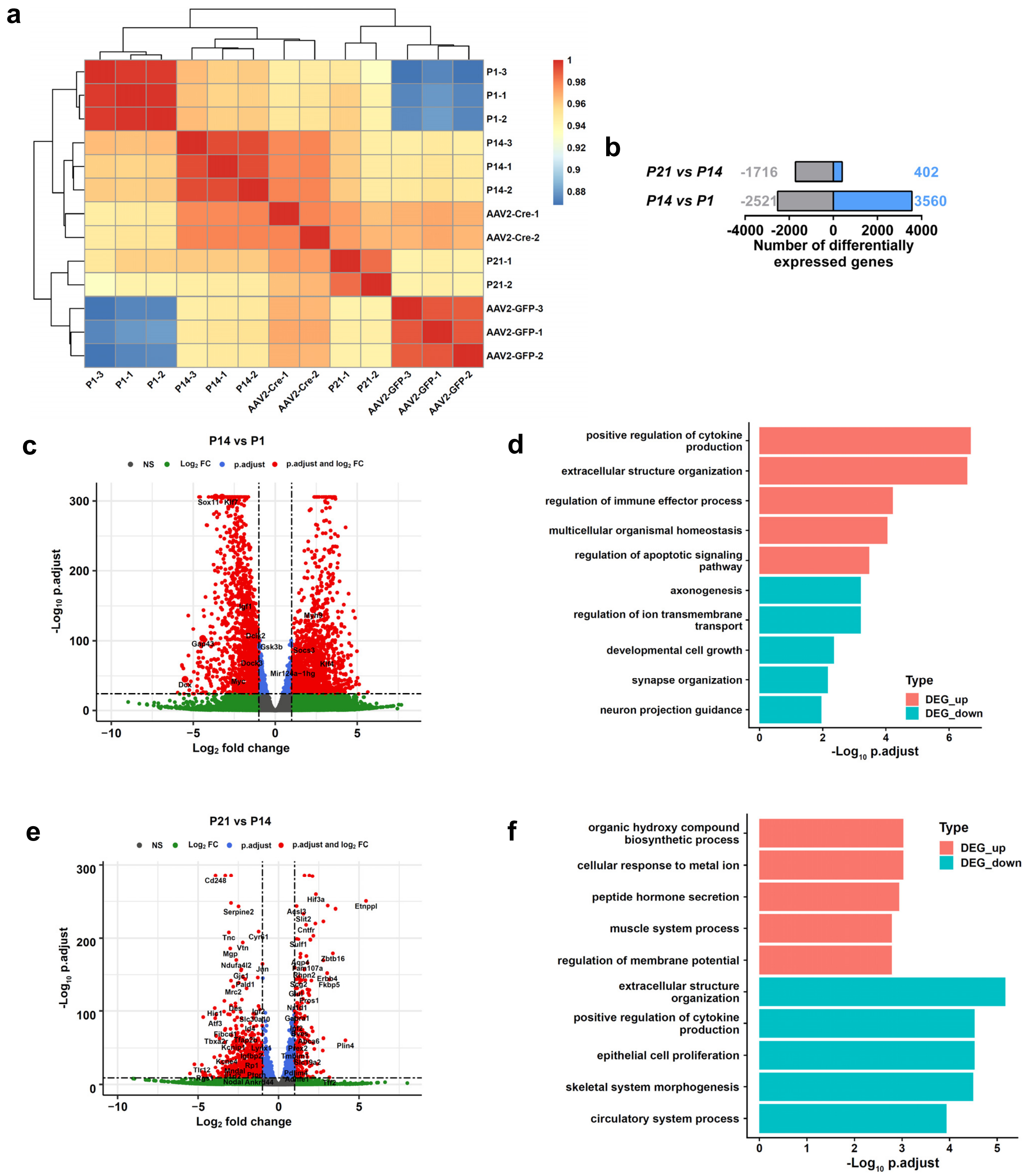

Extended Data Fig. 6 | Axon growth competence reduces during neuronal maturation, Related to Fig 7.

(a) Pearson's correlation analysis of normalized expression levels of all biological samples, including different developmental stages (P1, P14 and P21), 3 days post-injury RGCs from wild type (AAV2-GFP) and Kdm6a KO (AAV2-Cre) mice.

(b) Number of DEGs during neuronal maturation at the threshold of absolute $\log_2$ Fold change > 1 and adjusted *p*-value < 0.05.

(c) Volcano plot showing differential gene expression between P14 and P1 RGCs. Positive or negative $\log_2$ Fold change indicates upregulation or downregulation in P14 RGCs relative to P1 RGCs, respectively. Black points (NS) indicate the genes with no significant change. Green points (Log_2 FC) indicate the genes with absolute $\log_2$ Fold change > 1. Blue points (p.adjust) indicate the genes with adjusted *p*-value < 0.05. Red points (p.adjust and $\log_2$ FC) indicate that the genes are considered significantly different if absolute $\log_2$ Fold change > 1 and adjusted *p*-value < 0.05; vertical and horizontal reference lines at respective values.

(d) Top 5 GO terms were generated separately for genes upregulated or downregulated (adjusted *p*-value < 0.05) by P14 RGCs relative to P1 RGCs.

(e) Volcano plot showing differential gene expression between P21 and P14 RGCs. Positive or negative $\log_2$ Fold change indicates upregulation or downregulation in P21 RGCs relative to P14 RGCs, respectively. Black points (NS) indicate the genes with no significant change. Green points (Log_2 FC) indicate the genes with absolute $\log_2$ Fold change > 1. Blue points (p.adjust) indicate the genes with adjusted *p*-value < 0.05. Red points (p.adjust and $\log_2$ FC) indicate that the genes are considered significantly different if absolute $\log_2$ Fold change > 1 and adjusted *p*-value < 0.05; vertical and horizontal reference lines at respective values.

(f) Top 5 GO terms were generated separately for genes upregulated or downregulated (adjusted *p*-value < 0.05) by P21 RGCs relative to P14 RGCs.

Extended Data Figure 7

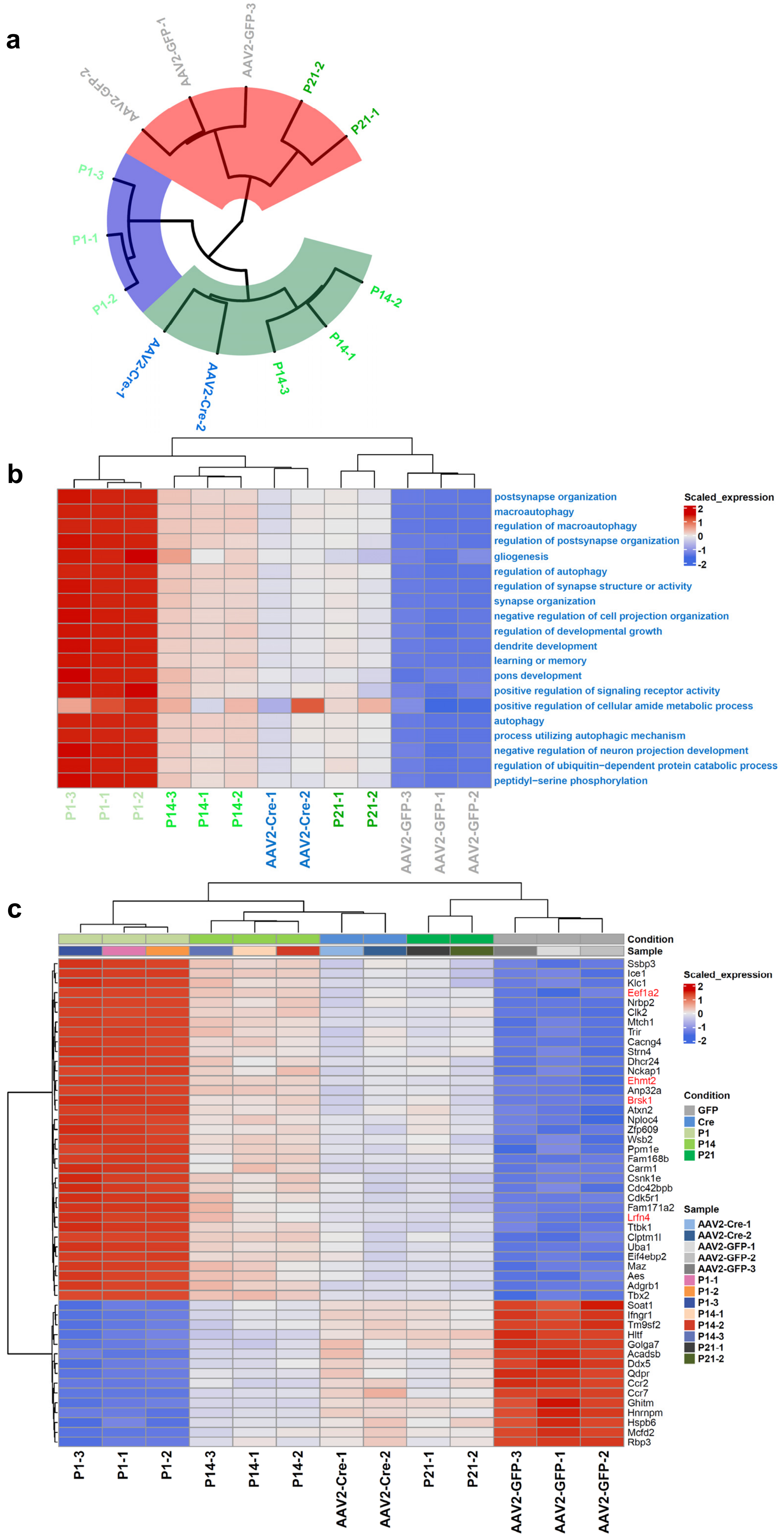

Extended Data Fig. 7 | Deleting *Kdm6a* in RGCs led to a youthful transcriptomic state, Related to Fig 7.

(a) Hierarchical clustering based on Euclidean distance of all samples. Circular dendrogram shows two major clusters, while P1, P14 and Kdm6a KO (AAV2-Cre) clustered together, P21 and wild type (AAV2-GFP) clustered separately.

(b) Heatmap of top 20 enriched GO terms for Dim 1 in Figure 7a. The Color intensities indicate the z-score of genes contributing to each GO term.

(c) Heatmap showing scaled expression of top 50 genes for Dim 1 in Figure 7a.

Extended Data Figure 8

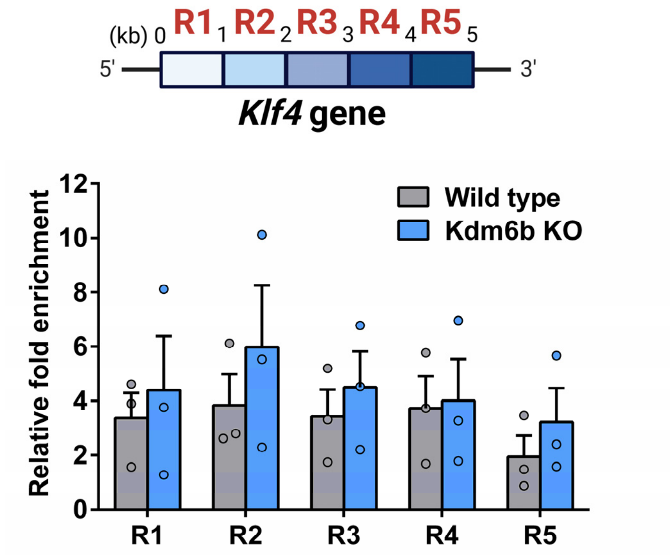

Extended Data Fig. 8 | Deletion of Kdm6b had no effect on *Klf4* gene H3K27me3 modification in RGCs, Related to Fig. 8.

Top: Schematic drawing of the 5 kilobase (kb) genomic regions (R1-R5) after TSS of the *Klf4* gene on chr4 were assayed in CUT&Tag-qPCR experiment using the antibody against H3K27me3 in the purified RGCs. In the H3K27me3 CUT&Tag-qPCR experiment, the genomic region R1-R5 were amplified from the purified RGCs to bind to H3K27me3. Bottom: CUT&Tag-qPCR results showing that deletion of Kdm6b (Kdm6b KO) had no effect on the interactions between H3K27me3 and specific regions of the *Klf4* gene (two tailed student's *t* tests, $p>0.05$; $n=3$ independent experiments for each condition). Data are represented as mean ± SEM.

Extended Data Figure 9

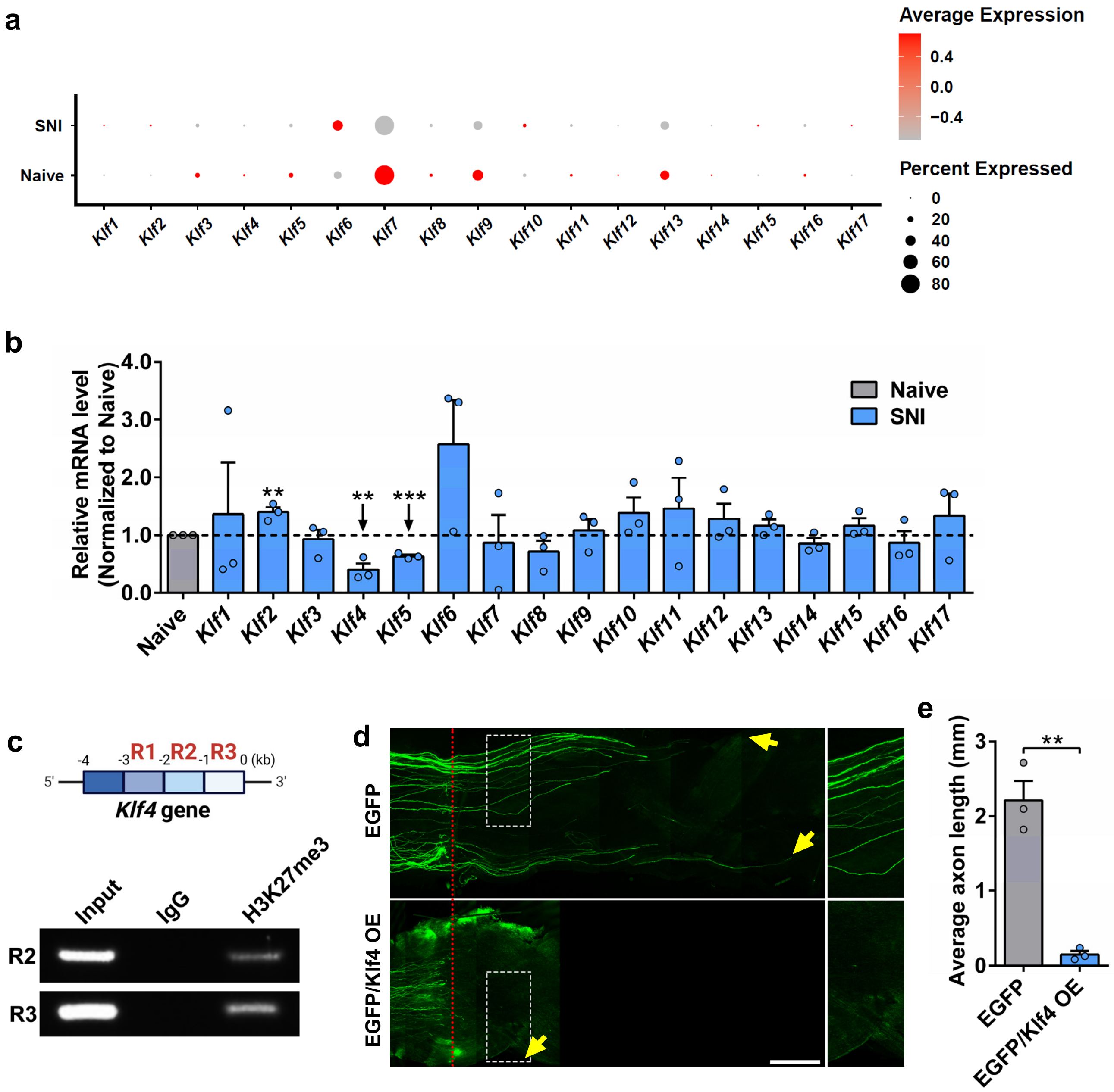

Extended Data Fig. 9 | Klf4 functions as a downstream target repressed by H3K27me3 to suppress axon regeneration in adult sensory neurons, Related to Fig. 8.

(a) Dot plot showing expression patterns of the Krüppel-like factor (Klf) family in DRG neurons after SNI. The plot was obtained from GEO: GSE154659 (uninjured: GSM4676534-537, SNI: GSM4676562-63). The size of each circle is proportional to the percentage of DRG neurons expressing *Klfs* and their intensity depicts the average transcript count within expressing DRG neurons.

(b) Changes in expression of *Klfs* in adult mouse DRG tissues upon SNI. Quantitative real-time PCR results showing significantly increased mRNA level of *Klf2* in DRG tissues 3 days post-SNI (one sample *t* test, p=0.0047; n=3 independent experiments for each condition), but significantly reduced mRNA level of *Klf4/5* (one sample *t* test, *Klf4*: p=0.0032; *Klf5*: p<0.0001; n=3 independent experiments for each condition).

(c) Top: Schematic drawing of the 3 kilobase (kb) genomic regions (R1-R3) before TSS of the *Klf4* gene on chr4 that were assayed in the ChIP experiment using the antibody against H3K27me3 in regenerating sensory neurons. Bottom: in the H3K27me3 ChIP experiment, the genomic region R2 and R3 were amplified from injured adult DRG tissues to bind to H3K27me3.

(d) Representative images of in vivo sensory axon regeneration in mice electroporated with either EGFP or EGFP+Klf4 overexpression (EGFP/Klf4 OE). The right column shows enlarged images of areas indicated by the dashed white boxes in the left column. The red dotted lines indicate the nerve crush sites. Yellow arrows indicate the distal ends of regenerating axons. Scale bar, 1 mm for the left column and 0.5 mm for the right column.

(e) Overexpressing *Klf4* in sensory neurons significantly blocked sensory axon regeneration in vivo (two tailed student's *t* tests, p=0.0015; EGFP; n= 3 mice, EGFP/Klf4 OE; n= 3 mice).

**, ***p<0.01, 0.001, respectively. Data are represented as mean ± SEM.
