# Supplementary material for: Roles of Kdm6a and Kdm6b in Regulation of Mammalian Neural Regeneration
Source: Adv Sci (Weinh). 2025 Feb 14;12(16):2405537. doi: 10.1002/advs.202405537 (PMC12021076; doi:10.1002/advs.202405537)
Supplement: Supplementary file 2 — Supporting Table [file ADVS-12-2405537-s002.docx]

Extended data table 1. List of primer sequences used in the current study.

| Gene | Sequence | Application |
| --- | --- | --- |
| *Kdm6a* | Forward: TAACCAAAGAGAGCAAGCCTTC | Gene expression |
|  | Reverse: GGACAGTTGGGTGGATGTTATT |  |
| *Kdm6b* | Forward: CTCAACTTGGGCCTCTTCTC | Gene expression |
|  | Reverse: ACGCAATCCTGCTCCCTCCC |  |
| *Klf1* | Forward: CGGCGAACTTTGGCACCTAAGA | Gene expression |
|  | Reverse: AGGAGCAGGCATAAGGCTTCTC |  |
| *Klf2* | Forward: CTCAGCGAGCCTATCTTGCC | Gene expression |
|  | Reverse: CACGTTGTTTAGGTCCTCATCC |  |
| *Klf3* | Forward: CCTCTCATGGTTTCCTTGTCGG | Gene expression |
|  | Reverse: CCTCTGTGGTTCAATTCCAGGC |  |
| *Klf4* | Forward: GTGCCCCGACTAACCGTTG | Gene expression |
|  | Reverse: GTCGTTGAACTCCTCGGTCT |  |
| *Klf5* | Forward: CCGGAGACGATCTGAAACACG | Gene expression |
|  | Reverse: GTTGATGCTGTAAGGTATGCCT |  |
| *Klf6* | Forward: GGAAGGTTGTGAGTGGCGTTTTG | Gene expression |
|  | Reverse: AGGTGGTCAGACCTGGAGAAAC |  |
| *Klf7* | Forward: GGAAGGATGCGAGTGGCGTTTT | Gene expression |
|  | Reverse: CGCAAGATGGTCAGACCTGGAG |  |
| *Klf8* | Forward: CCTGAAAGCTCACCGCAGAATC | Gene expression |
|  | Reverse: CGTGTGCTTACGGAAATGGCGA |  |
| *Klf9* | Forward: GCCGCCTACATGGACTTCG | Gene expression |
|  | Reverse: GCCGTTCACCTGTATGCAC |  |
| *Klf10* | Forward: CACACCAGTGTCTGATACCTCC | Gene expression |
|  | Reverse: TAGATGGCGCTGATGCAGTCAG |  |
| *Klf11* | Forward: GCTCATCTTCGCACTCACACAG | Gene expression |
|  | Reverse: TCTTCTCTCCCGTGTGAGTCCT |  |
| *Klf12* | Forward: CCTTTCCATAGCCAGAGCAGTAC | Gene expression |
|  | Reverse: TGGCGTCTTGTGCTCTCAATGC |  |
| *Klf13* | Forward: CCTGGCCTCAGACAAAGGG | Gene expression |
|  | Reverse: ATTTCCCGTAAACTTTCTCGCA |  |
| *Klf14* | Forward: TACCGAAGGAGGCAGATTACGC | Gene expression |
|  | Reverse: GTCGAGCCAATCACAGGAGAAG |  |
| *Klf15* | Forward: GAGACCTTCTCGTCACCGAAA | Gene expression |
|  | Reverse: GCTGGAGACATCGCTGTCAT |  |
| *Klf16* | Forward: ATCCTGGCCGATCTGAGAGG | Gene expression |
|  | Reverse: GTGCGAAGACTTGTAATAGGCT |  |
| *Klf17* | Forward: CCTTGTGAGTCACCAACGCAAAC | Gene expression |
|  | Reverse: CCGTTTGTGTCGTCCAAGCTCA |  |
| *Klf18* | Forward: CCTGTCTCGGGACTGCAAAA | Gene expression |
|  | Reverse: CCTGAGAAAAGCTGGCTGGA |  |
| *Pten* | Forward: AGACCATAACCCACCACAGC | Gene expression |
|  | Reverse: GTCCGTCCCTTTCCAGCTTT |  |
| *Cdk5r1* | Forward: CTGTCCCTATCCCCCAGCTAT  Reverse: GGCAGCACCGAGATGATGG | Gene expression |
| *Gpc2* | Forward: GACATGGGAGTGAGGCAAAGG  Reverse: CCTGAGGACAGACTTGAAGGT | Gene expression |
| *Rabgef1* | Forward: ATGAGCCTGAAGTCCGAACG  Reverse: GCCTTGTGGTACTCCTCCCT | Gene expression |
| *Tnfsf13* | Forward: CTTTCGGTTGCTCTTTGGTTG  Reverse: CGACAGCACAAGTCACAGC | Gene expression |
| *Pias3* | Forward: GAAGGAGGCATCAGAGGTTTG  Reverse: TAGACAGGAAATCACTGCCCA | Gene expression |
| *Gapdh* | Forward: AGGTCGGTGTGAACGGATTTG | Gene expression |
|  | Reverse: TGTAGACCATGTAGTTGAGGTCA |  |
| *R1* 3kb upstream | Forward: CCCTTTCCTCTGCCTAGCTT | ChIP-PCR |
|  | Reverse: CTGGGACTGTAGGTGCCAAT |  |
| *R2* 2kb upstream | Forward: CGAGTGCCTTTCTTCAGTCC | ChIP-PCR |
|  | Reverse: CAAAAACCGTGAGTGTGGTG |  |
| *R3* 1kb upstream | Forward: TTGAGATCCCTGGTGAAAGG | ChIP-PCR |
|  | Reverse: GCGCCTGCTATGAAAGAGAG |  |
| *R1* 1kb downstream | Forward: CCCAAAGGATCCCATGCCAT  Reverse: TCTCGAAATAACAAAGAACGCAC | CUT&Tag-qPCR |
| *R2* 2kb downstream | Forward: GATAATGGAGAGAGGGGACTTGTG  Reverse: AGTCCCGAGGAACTGCTGAA | CUT&Tag-qPCR |
| *R3* 3kb downstream | Forward: GTTAATTGTAGCACCCTGTTGCC  Reverse: ACCTTGGTGTTAGCTGAGATTGC | CUT&Tag-qPCR |
| *R4* 4kb downstream | Forward: GGATTCAATATAAACCGGCGATG  Reverse: ACCTACTTATCTGCCTTGCTGA | CUT&Tag-qPCR |
| *R5* 5kb downstream | Forward: TCTCTCGCATACGCACGCA  Reverse: CTTTCATAGCAGGCGCGGAA | CUT&Tag-qPCR |
